# Editorial: Psychological determinants of entrepreneurial intentions and behaviors; contributions of Robert D. Hisrich to the field of entrepreneurship

**DOI:** 10.3389/fpsyg.2023.1196889

**Published:** 2023-05-11

**Authors:** Boštjan Antončič, Jasna Auer Antončič

**Affiliations:** ^1^School of Economics and Business, University of Ljubljana, Ljubljana, Slovenia; ^2^Faculty of Management, University of Primorska, Koper, Slovenia

**Keywords:** psychology, personality, entrepreneurship, entrepreneurial intentions, behaviors

## Introduction

Professor Robert D. Hisrich, Ph.D., the co-editor of this Research Topic, passed away in February 2023, so we decided to dedicate this editorial to him and his work and describe articles in this Research Topic (a Research Topic on *Psychological determinants of entrepreneurial intentions and behaviors*) considering extensions of his research.

## Robert D. Hisrich

Robert D. Hisrich, born in 1944, was a wonderful person, the best mentor, the best professor, and one of the greatest scholars of all time in the field of entrepreneurship. We are very grateful to him for helping, teaching, and inspiring us and numerous academics and practitioners globally. He will live in our hearts and memories forever. His works will be read and cited also by future generations.

Professor Hisrich was the Bridgestone Chair of International Marketing & Director of The Global Management Center and International Programs at Kent State University, Kent, Ohio. He also held visiting or honorary professor positions at universities in six different countries outside the United States (e.g., the longest cooperation with the School of Economics and Business at the University of Ljubljana, Slovenia, since 1995). His past positions included, e.g., Garvin Professor of Global Entrepreneurship and Director, Walker Center for Global Entrepreneurship, Thunderbird School of Global Management, Phoenix, Arizona; Malachi Mixon III Chair in Entrepreneurial Studies and Professor and Chair of Strategy Division and Entrepreneurship Division, Weatherhead School of Management, Case Western Reserve University, Cleveland, Ohio; Chair of the Entrepreneurship Division of the Academy of Management; Bovaird Chair of Entrepreneurial Studies and Private Enterprise and Professor of Marketing, College of Business Administration, University of Tulsa, Tulsa, Oklahoma; Associate Professor of Marketing, Graduate School of Management, Boston College, Chestnut Hill, Massachusetts; Adjunct Professor of Marketing and Technology, Innovation Center, Massachusetts Institute of Technology, Cambridge, Massachusetts; Faculty Associate Small Business Development Center; Director, Small Business Institute; Director, H&B Associates, a marketing and management-consulting firm. He authored or co-authored 37 books and more than 300 articles on entrepreneurship, international business, venture capital, management, and marketing.

His two most influential books were Entrepreneurship (Hisrich et al., 2020), 11 published editions and the 12th in preparation, and The Woman Entrepreneur (Hisrich and Brush, 1985). The Entrepreneurship books broadened the horizons of entrepreneurship, starting businesses, and managing small and medium-sized enterprises (SMEs) and large corporations of numerous entrepreneurs, businesspeople, students, government officials, and others. It has been read globally in English and other languages. The Woman Entrepreneur book highlights the importance of women in entrepreneurship and their characteristics. The famous definition of entrepreneurship states (Hisrich et al., 2005, p. 8; Hisrich et al., 2007, p. 576) the following: “Entrepreneurship is the process of creating something new with value by devoting the necessary time and effort, assuming the accompanying financial, psychic, and social risks, and receiving the resulting rewards”.

His most influential articles—the top 30 publications with 10 or more citations in the Web of Science—are displayed in Table 1. His articles were the most influential in 10 areas (Table 2), among which the top 3 were business, management, and economics, followed by psychology (applied and multidisciplinary). Specifically, the topics covered by his works were intrapreneurship, venture capital, female entrepreneurship, psychological aspects of entrepreneurship, SME internationalization, ethics of entrepreneurs, entrepreneurship in transition economies, new venture creation, and marketing.

**Table 1:** Top 30 publications in the Web of Science (sorted by the number of citations).

**Rank**	**Title**	**References**	**Journal**	**Citations**
1	Intrapreneurship: Construct refinement and cross-cultural validation	Antoncic and Hisrich, 2001	Journal of Business Venturing	581
2	Toward a model of venture capital-investment decision-making	Fried and Hisrich, 1994	Financial Management	324
3	Israeli women entrepreneurs: An examination of factors affecting performance	Lerner et al., 1997	Journal of Business Venturing	211
4	Entrepreneurship Intrapreneurship	Hisrich, 1990	American Psychologist	200
5	Entrepreneurship research and practice - A call to action for psychology	Hisrich et al., 2007	American Psychologist	174
6	Human capital and SME internationalization: A structural equation modeling study	Ruzzier et al., 2007b	Canadian Journal of Administrative Sciences -Revue Canadienne des sciences de l administration	154
7	The female entrepreneur - A career development perspective	Bowen and Hisrich, 1986	Academy of Management Review	153
8	The venture capitalist - A relationship investor	Fried and Hisrich, 1995	California Management Review	131
9	Strategy and the board of directors in venture capital-backed firms	Fried et al., 1998	Journal of Business Venturing	129
10	How venture capital firms differ	Elango et al., 1995	Journal of Business Venturing	119
11	Intuition in venture capital decisions – An exploratory-study using a new technique	Hisrich and Jankowicz, 1990	Journal of Business Venturing	104
12	Entrepreneurs' creativity and firm innovation: The moderating role of entrepreneurial self-efficacy	Ahlin et al., 2014a	Small Business Economics	93
13	Ethics and entrepreneurs - An international comparative study	Bucar et al., 2003	Journal of Business Venturing	85
14	Product innovation and firm performance in transition economies: A multi-stage estimation approach	Ramadani et al., 2019a	Technological Forecasting and Social Change	66
15	Entrepreneurship in the Soviet-union and post-socialist Russia	Ageev et al., 1995	Small Business Economics	48
16	Perceived risk in store selection	Hisrich et al., 1972	Journal of Marketing Research	42
17	Intrapreneurship strategy for internal markets–Corporate, non-profit and government institution cases	Nielsen et al., 1985	Strategic Management Journal	41
18	Tthe Russian entrepreneur	Hisrich and Grachev, 1993	Journal of Business Venturing	40
19	Beekeeping as a family artisan entrepreneurship business	Ramadani et al., 2019b	International Journal of Entrepreneurial Behavior and Research	30
20	Risk-taking propensity and entrepreneurship: The role of power distance	Auer Antoncic et al., 2018	Journal of Enterprising Culture	25
21	The internationalization of SMEs: Developing and testing a multi-dimensional measure on Slovenian firms	Ruzzier et al., 2007a	Entrepreneurship and Regional Development	25
22	Technological innovativeness and firm performance in Slovenia and Romania	Antoncic et al., 2007	Post-Communist Economies	24
23	Selecting superior segmentation correlate	Hisrich and Peters, 1974	Journal of Marketing	24
24	Entrepreneurial stressors as predictors of entrepreneurial burnout	Wei et al., 2015	Psychological Reports	19
25	Exploring the moderating effects of absorptive capacity on the relationship between social networks and innovation	Ahlin et al., 2014b	Journal of East-European Management Studies	18
26	Manufacturing entrepreneurs – An empirical-study of the correlates of employment growth in the Tulsa MSA and rural East Texas	Box et al., 1994	Journal of Business Venturing	17
27	Entrepreneurial openness: Concept development and measure validation	Slavec et al., 2017	European Management Journal	13
28	The mediating role of corporate entrepreneurship for externam environment effects on performance	Kearney et al., 2013	Journal of Business Economics and Management	13
29	New business formation through the enterprise development center - A model for new venture creation	Hisrich, 1988	IEEE Transactions on Engineering Management	11
30	Executive advertisers views on comparison advertising	Hisrich, 1983	Sloan Management Review	10

Source: Web of Science (2023).

**Table 2:** Impact–top 10 categories.

**Rank**	**Category**	**Citations (impacted publications)**	**% of 2,130 citations**
1	Business	1,096	51.5%
2	Management	894	42.0%
3	Economics	268	12.6%
4	Psychology applied	91	4.3%
5	Business finance	84	3.9%
6	Educational research	59	2.8%
7	Environmental studies	59	2.8%
8	Green sustainable science technology	57	2.7%
9	Environmental sciences	56	2.6%
10	Psychology multidisciplinary	54	2.5%

Source: Web of Science (2023).

## Psychological determinants of entrepreneurial intentions and behaviors

Current researchers in the field of entrepreneurship are standing on the shoulders of giants—the past researchers (Robert D. Hisrich and others), who made prior developments to theories and concepts. For example, Hisrich (1990) presented the features of entrepreneurship and intrapreneurship from a psychological perspective. This Research Topic covers topics in entrepreneurial psychology with a range of original research articles from different research areas and perspectives that examine personality determinants of entrepreneurship and used data from various countries (Australia, China, Malaysia, Saudi Arabia, Slovenia, Spain, South Africa, and the United States of America). Hisrich et al. (2007, p. 575) motivated researchers within entrepreneurial psychology “to develop theory and undertake empirical research focusing on five key topic areas: the personality characteristics of entrepreneurs, the psychopathology of entrepreneurs, entrepreneurial cognition, entrepreneurship education, and international entrepreneurship”. This Research Topic focused mostly on the personality characteristics of entrepreneurs.

Antoncic (2020) proposed directions for future research: “Personality correlates of entrepreneurs need to be researched in cross-cultural comparative studies and in studies that include sociological elements, which may be related to the formation of the personality or behavior of individuals with entrepreneurial intentions and entrepreneurs and intrapreneurs. The personality differences between entrepreneurs and intrapreneurs also need to be investigated in greater detail in future research. Theoretical models need to be, on one hand, more comprehensive (with many different correlates) and, on the other hand, more detailed (with many sub-dimensions and facets in quantitative research, as well as with more in-depth explanations of phenomena in qualitative research).” This Research Topic incorporated these directions well since it includes the following: a cross-cultural study (Soltwisch et al.); studies with sociological elements (Antončič and Auer Antončič; Bai et al.; Guo et al.; Qiu et al.); studies including intrapreneurship elements (Thomran et al.; Soltwisch et al.); studies with more comprehensive theoretical models (Antončič and Auer Antončič; Bergner et al.; Guo et al.; Heinemann at al.; Soltwisch et al.; Volery and Mattes); a case study (Bai et al.); and studies examining social (Bergner et al.; Soltwisch et al.) and serial (Bai et al.) entrepreneurial intentions.

The key personality correlates of entrepreneurship were summarized in past research (e.g., Antoncic, 2020): the Big Five personality factors, entrepreneurial self-efficacy, need for independence, risk-taking propensity, internal locus of control, and need for achievement. Most of these elements are included in this Research Topic, with the addition of other personality and non-personality correlates of entrepreneurship: (1) Volery and Mattes: the Big Five personality, education, necessity entrepreneurs, and self-employment survival/exit; (2) Antončič and Auer Antončič: all psychology correlates from Antoncic (2020), sociological elements (family business background, local business support background), and start-up intentions and actions; (3) Heinemann et al.: epistemic curiosity, openness to experience, entrepreneurial alertness, and entrepreneurship (intention and orientation); (4) Hamzah and Othman: internal locus of control, external locus of control, and entrepreneurial competency; (5) Qiu et al.: faultline configurations and the entrepreneurial team performance; (6) Thomran et al.: autonomy, risk-taking, proactiveness, innovativeness, competitive aggression, cost advantage, and differentiation advantage; (7) Guo et al.: improvisation, entrepreneurial self-efficacy, policy support for entrepreneurship, and entrepreneurial intention; (8) López-Núñez et al.: self-efficacy, emotional intelligence, the Big Five personality, and entrepreneurial intention; (9) Soltwisch et al.: decision styles, cultural orientation, entrepreneurial intention, and social entrepreneurial intention; (10) Bergner et al.: personality, cognition, entrepreneurial exposition, and social entrepreneurial intention; (11) Bai et al.: entrepreneurial expectations, identification and evaluation of opportunities, entrepreneurial failure learning, entrepreneurial cognitive schema, behavioral addiction tendency, emotional perception and motivation, entrepreneurial experience, environmental conditions, financial conditions, demographic factors, and serial entrepreneurial intention.

## Conclusion

Articles in this Research Topic—the Research Topic on *Psychological determinants of entrepreneurial intentions and behaviors*—have expanded the prior knowledge base about several psychological determinants of entrepreneurship and provided recommendations for research and practice.

## Author contributions

BA and JA contributed to the conception and design of the article, wrote the first draft of the manuscript, and wrote all sections. Both authors contributed to the manuscript's revision and read and approved the submitted version.
